# Norovirus Replication in Human Intestinal Epithelial Cells Is Restricted by the Interferon-Induced JAK/STAT Signaling Pathway and RNA Polymerase II-Mediated Transcriptional Responses

**DOI:** 10.1128/mBio.00215-20

**Published:** 2020-03-17

**Authors:** Myra Hosmillo, Yasmin Chaudhry, Komal Nayak, Frederic Sorgeloos, Bon-Kyoung Koo, Alessandra Merenda, Reidun Lillestol, Lydia Drumright, Matthias Zilbauer, Ian Goodfellow

**Affiliations:** a Division of Virology, Department of Pathology, University of Cambridge, Cambridge, United Kingdom; b Department of Paediatrics, University of Cambridge, Cambridge, United Kingdom; c Wellcome Trust-Medical Research Council Stem Cell Institute, University of Cambridge, Cambridge, United Kingdom; d Institute of Molecular Biotechnology of the Austrian Academy of Sciences (IMBA), Vienna Biocenter (VBC), Vienna, Austria; e Department of Medicine, Addenbrooke’s Hospital, University of Cambridge, Cambridge, United Kingdom; University of Pittsburgh School of Medicine

**Keywords:** intestine, organoid, interferons, mucosal pathogens, noroviruses

## Abstract

Human noroviruses (HuNoV) are a leading cause of viral gastroenteritis worldwide and a significant cause of morbidity and mortality in all age groups. The recent finding that HuNoV can be propagated in B cells and mucosa-derived intestinal epithelial organoids (IEOs) has transformed our ability to dissect the life cycle of noroviruses. Using transcriptome sequencing (RNA-Seq) of HuNoV-infected intestinal epithelial cells (IECs), we have found that replication of HuNoV in IECs results in interferon (IFN)-induced transcriptional responses and that HuNoV replication in IECs is sensitive to IFN. This contrasts with previous studies that suggested that the innate immune response may play no role in the restriction of HuNoV replication in immortalized cells. We demonstrated that inhibition of Janus kinase 1 (JAK1)/JAK2 enhanced HuNoV replication in IECs. Surprisingly, targeted inhibition of cellular RNA polymerase II-mediated transcription was not detrimental to HuNoV replication but instead enhanced replication to a greater degree than blocking of JAK signaling directly. Furthermore, we demonstrated for the first time that IECs generated from genetically modified intestinal organoids, engineered to be deficient in the interferon response, were more permissive to HuNoV infection. Taking the results together, our work revealed that IFN-induced transcriptional responses restrict HuNoV replication in IECs and demonstrated that inhibition of these responses mediated by modifications of the culture conditions can greatly enhance the robustness of the norovirus culture system.

## Abstract (precis)

Noroviruses are a major cause of gastroenteritis worldwide, and yet the challenges associated with their growth in culture have greatly hampered the development of therapeutic approaches and have limited our understanding of the cellular pathways that control infection. Here, we show that human intestinal epithelial cells, which represent the first point of entry of human noroviruses into the host, limit virus replication by induction of innate responses. Furthermore, we show that modulating the ability of intestinal epithelial cells to induce transcriptional responses to HuNoV infection can significantly enhance human norovirus replication in culture. Collectively, our findings provide new insights into the biological pathways that control norovirus infection but also identify mechanisms that enhance the robustness of norovirus culture.

## INTRODUCTION

The induction of the host innate response plays an essential role in the suppression of pathogen infection. The synthesis of interferons (IFN) and the subsequent signaling cascades that lead to the induction of IFN-stimulated genes (ISGs) determine the outcome of viral infection (1, 2). An understanding of the mechanisms underlying the interplay between pathogens and innate immune responses is vital to understanding viral pathogenesis and can greatly aid the identification of potential therapeutic and/or preventive strategies.

Human noroviruses (HuNoV) are widely recognized as the leading cause of viral gastroenteritis worldwide (3). Noroviruses are classified into at least seven genogroups based on the sequence of the major capsid protein VP1 and regions within ORF1 (3–5). HuNoVs belong to one of three norovirus genogroups (GI, GII, and GIV), which are further divided into >25 genetic clusters or genotypes (6–8). Epidemiological studies have revealed that over 75% of confirmed human norovirus infections are associated with HuNoV GII (9, 10). While norovirus gastroenteritis typically results in an acute and self-limiting disease, the socioeconomic impact in both developed and developing countries is estimated to be more than $60.3 billion per annum (11). HuNoV infection is particularly severe and prolonged in immunocompromised patients, including young children, the elderly, or patients receiving treatment for cancer. In these cases, infections can last months to years (12, 13).

Our understanding of the molecular mechanisms that control HuNoV infection has been limited by the lack of robust culture systems that facilitate detailed analysis of the viral life cycle. As a result, murine norovirus (MNV) and other members of the *Caliciviridae* family of positive-sense RNA viruses, such as feline calicivirus (FCV) and porcine sapovirus (PSaV), are often used as surrogate models (14–17). MNV, FCV, and PSaV can all be efficiently cultured in immortalized cells and are amenable to reverse genetics (16–20). These model systems have been critical to understanding many aspects of the life cycle of members of the *Caliciviridae* (15).

Recent efforts have led to the establishment of two HuNoV culture systems based on immortalized B cells (21, 22) and on intestinal epithelial cells (IECs) generated from biopsy-derived human intestinal epithelial organoids (IEOs) (23). While authentic replication of HuNoV can be observed in both the B-cell-based and IEC-based culture systems, repeated long-term passage of HuNoV and generation of high-titer viral stocks are not possible, suggesting that replication is restricted in some manner. In the current study, we sought to better understand the cellular response to HuNoV infection and to identify pathways that restrict HuNoV replication in organoid-derived IECs. Using transcriptome sequencing (RNA-Seq), we observed that HuNoV infection of IECs resulted in an interferon-mediated antiviral transcriptional response. To our knowledge, we show for the first time that HuNoV replication in IECs is sensitive to both type I and type III interferon and that HuNoV replication is restricted by virus-induced innate responses. Pharmacological inhibition of the interferon response or genetic modification of organoids to prevent the activation of the interferon response significantly improved HuNoV replication in IECs. Furthermore, we show that ongoing HuNoV replication was enhanced by the inhibition of RNA polymerase II (Pol II)-mediated transcription. Overall, this work provides new insights into the cellular responses to HuNoV infection of the gut epithelium and identifies modifications to the HuNoV culture system that significantly enhance its utility.

## RESULTS

### Human norovirus replicates productively in differentiated intestinal epithelial cells from the human proximal and distal small bowel.

**FIG 1:**
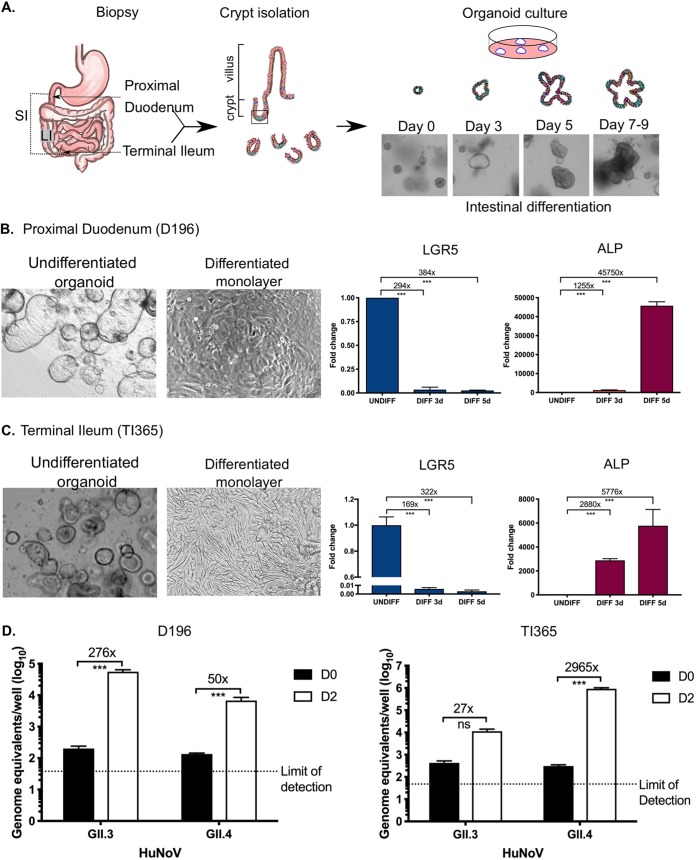
Overview of the human norovirus culture system. (A) Schematic of the intestinal crypt isolation procedure leading to the production of intestinal organoids. Following isolation by biopsy, crypts were plated into Matrigel as described in the text and imaged by light microscopy. (B and C) Differentiation of intestinal organoids from the duodenum (B) and terminal ileum (C) into monolayers of intestinal epithelial cells (IECs) is accompanied by loss of the stem cell marker LGR5 and increased intestinal alkaline phosphatase (ALP) expression. Intestinal organoid lines were plated onto collagen-coated plates as described in the text, and the relative levels of LGR5 and ALP were quantified by RT-qPCR. Expression levels are shown relative to the results seen with the undifferentiated cells extracted on day 0 of plating. (D) Infection of differentiated IECs from the duodenum (D196) and terminal ileum (TI365) with two clinical isolates of human norovirus (GII.3 and GII.4). Infection was assessed by the quantification of absolute viral RNA levels by RT-qPCR, and the results are shown as both absolute values (D) and fold change values representing the increases in viral RNA levels seen in comparisons of the results from day 0 (D0) to those from day 2 (D2). All experiments were performed at least two independent times, and results are expressed as means ± standard errors of the means (SEM) from duplicate and triplicate samples analyzed in technical duplicate. Statistically significant values are represented as follows: *, *P* ≤ 0.05; **, *P* ≤ 0.01; ***, *P* ≤ 0.001; ****, *P* ≤ 0.0001.

Building on previous studies reporting the replication of HuNoV in IECs, we set out to better understand the cellular response to HuNoV infection and to identify pathways that restrict HuNoV replication in IECs. We established IEO cultures using mucosal biopsy specimens obtained from several gut segments of the small intestine, the proximal duodenum, and terminal ileum. Given the importance of fucosyltransferase expression on HuNoV susceptibility (24–26), lines were established from FUT2-positive individuals. Intestinal crypt cells were isolated and used to generate small IEOs (Fig. 1A). The three-dimensional (3D) organoid structures were allowed to self-organize and differentiate within Matrigel by the use of optimized proliferation medium as described previously (27) (see Table S1 in the supplemental material). The established organoid lines were typically cultured for 7 to 9 days and expanded at a passage ratio of 1:2 or 1:3. As expected, the intestinal organoids initially formed small cystic structures with a central lumen, lined with epithelial cells, during the first 3 days of culture (Fig. 1A). By day 5 (D5), more-convoluted structures formed, the nature of which differed from line to line (Fig. 1A).

10.1128/mBio.00215-20.3 TABLE S1 Supplier information corresponding to the composition of the organoid growth and differentiation media used for the culture of mucosa-derived intestinal organoids in this study. Download Table S1, XLSX file, 0.01 MB. Copyright © 2020 Hosmillo et al. 2020 Hosmillo et al. This content is distributed under the terms of the Creative Commons Attribution 4.0 International license.

To assess the replication efficiency of HuNoV replication in differentiated IECs, 7-to-9-day-old organoids were plated onto collagen-coated plates, and then Wnt and RSpo were removed to drive differentiation. To examine the degree of differentiation and to confirm the presence of enterocytes in the monolayers, we examined the mRNA levels of LGR5 and alkaline phosphatase (ALP) in IEC monolayers generated from both duodenum and ileum (Fig. 1B and C). As shown in Fig. 1B, the levels of LGR5 mRNA in proximal duodenum decreased by ∼294-fold to 384-fold, whereas ALP mRNA levels increased by 1,200-fold to 45,000-fold following the removal of Wnt and RSpo. Similarly, differentiation of IECs derived from the terminal ileum was confirmed by an increase in ALP mRNA levels and a concomitant decrease in LGR5 levels (Fig. 1C). These results confirmed that the IEC monolayers had undergone differentiation and confirmed the presence of enterocytes in the differentiated monolayers.

To assess HuNoV replication in the human IEC monolayers, filtered stool samples containing genogroup II HuNoV strains were inoculated onto differentiated monolayers generated from either duodenum-derived or terminal ileum-derived intestinal organoids. Following a 2-h adsorption period, the inoculum was removed by washing and the monolayers were maintained in differentiation media with the bile acid glycochenodeoxycholic acid (GCDCA) for 2 days. While previous observations indicated that some strains of HuNoV do not require bile acids for infection, GCDCA was included to maintain a physiologically relevant environment and to control for any effect of bile acids on gene expression. Replication of HuNoV was then assessed by comparing the viral RNA levels present in cultures at 2 h postinfection (h pi) (day 0 [D0]) to those present at 48 h postinfection (day 2 [D2]). In duodenal IEC monolayers, the viral RNA levels of both GII.3 and GII.4 HuNoV strains increased by ∼1.5 to 2 log_10_ over the 2-day period (Fig. 1D). Similar levels of viral replication were observed in IEC monolayers derived from terminal ileum organoids, resulting in ∼1.3 to 3.5 log_10_ increases in viral RNA levels (Fig. 1D).

### Norovirus infection of human intestinal epithelial cells induces the innate immune response.

The development of a stem cell-derived culture system for HuNoV provides the first opportunity to characterize the cellular pathways that restrict norovirus replication at its primary site of entry into the host, namely, the gut epithelium (23). While a previous report suggested that replication of HuNoV did not induce a robust interferon response in immortalized cells (28), inefficient replication and the presence of mutations commonly found in cell lines that compromise their ability to respond to viral infection may have confounded that observation. While there are limited examples in the literature, there is evidence that natural HuNoV infection results in the production of proinflammatory and anti-inflammatory cytokines (29). We also recently reported that HuNoV replication in zebrafish also resulted in a measurable innate response (30).

**FIG 2:**
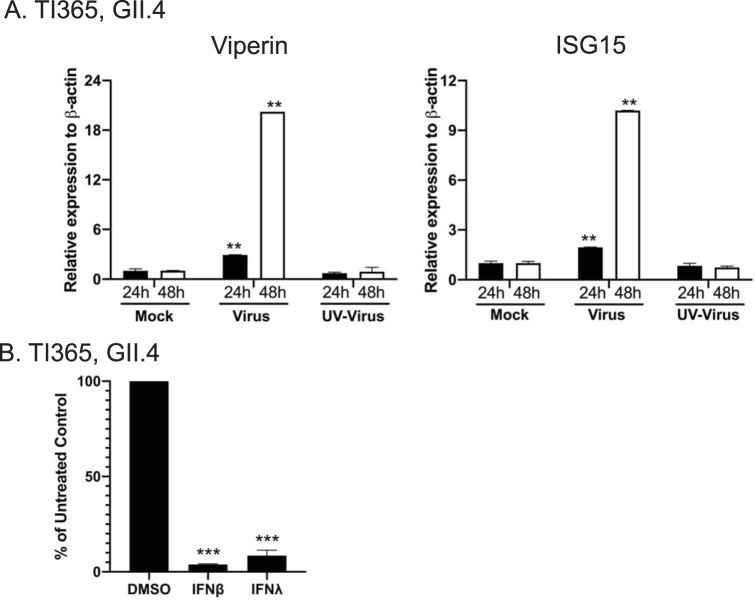
Human norovirus infection of intestinal epithelial cells induces interferon-stimulated genes and is sensitive to type I and type III interferon. (A and B) The levels of two interferon-stimulated genes, the viperin (A) and ISG15 (B) genes, following norovirus infection of differentiated intestinal epithelial cells from the terminal ileum (TI365), were assessed at 24 and 48 h postinfection. Monolayers were either mock infected or infected with active GII.4 human norovirus or UV-inactivated GII.4 human norovirus. Relative levels of gene expression were assessed by RT-qPCR and are shown in comparison to β-actin expression levels. (C) GII.4 human norovirus infection of terminal ileum is sensitive to type I interferon (IFN-β) and type III interferon (IFN-λ). Differentiated IEC monolayers were either mock treated or treated with recombinant IFN for 18 h prior to infection with GII.4 HuNoV. Viral RNA levels at 2 days postinfection were then quantified by RT-PCR, and the increased viral RNA levels were expressed as a percentage of those seen with the untreated control. All experiments were performed at least two independent times, and results are expressed as means ± SEM from duplicate and triplicate samples analyzed in technical duplicate. Statistically significant values are represented as follows: *, *P* ≤ 0.05; **, *P* ≤ 0.01; ***, *P* ≤ 0.001; ****, *P* ≤ 0.0001.

To examine the effect of HuNoV replication on the stimulation of the IFN-induced innate response, we initially assessed the mRNA levels of two candidate interferon-stimulated genes (ISGs), the human viperin and ISG 15 genes, in mock-infected, active HuNoV-infected, and UV-inactivated HuNoV-infected epithelial cells. Viperin and ISG15 mRNA levels increased significantly following HuNoV infection only (Fig. 2A). To confirm that the induction was specifically caused by active HuNoV replication, UV-inactivated HuNoV stool filtrates were used alongside to control for nonspecific effects. In contrast to the results seen with live virus-inoculated IEC monolayers, induction of viperin and ISG15 was not seen in cells infected with UV-inactivated virus or in mock-inoculated cells, confirming that the induction was virus specific (Fig. 2A). These results indicate for the first time that active replication of HuNoV in IECs readily stimulates the interferon-induced innate response.

To examine whether HuNoV replication in IECs was in fact sensitive to IFN and therefore, by extension, likely restricted by the virus-induced response, we examined the effect of IFN pretreatment on the replication of HuNoV GII.4 in IECs. The addition of either IFN-β1 or IFN-λ1 and IFN-λ2 (IFN-λ1/2) had an inhibitory effect on GII.4 HuNoV replication in IECs derived from terminal ileum organoids. This result confirms that HuNoV infection of IECs is sensitive to antiviral effects of type I interferon (IFN-β1) or type III interferon (IFN-λ1/2) (Fig. 2B). We therefore hypothesized that the HuNoV-induced transcriptional responses might restrict HuNoV replication in IECs.

### Human norovirus replication in intestinal epithelial cells activates the IFN-induced JAK/STAT signaling pathway.

The impact of norovirus infection on host gene expression in IECs and on the magnitude of the IFN response during HuNoV infection was examined by performing RNA-Seq analysis of infected IEC monolayers. IECs from two independent terminal ileum-derived organoid cultures were mock infected, infected with a patient-derived active GII.4 HuNoV strain, or infected with a UV-inactivated sample of the same inoculum. Two days postinfection, total cellular RNA was extracted and processed for RNA-Seq analysis.

**FIG 3:**
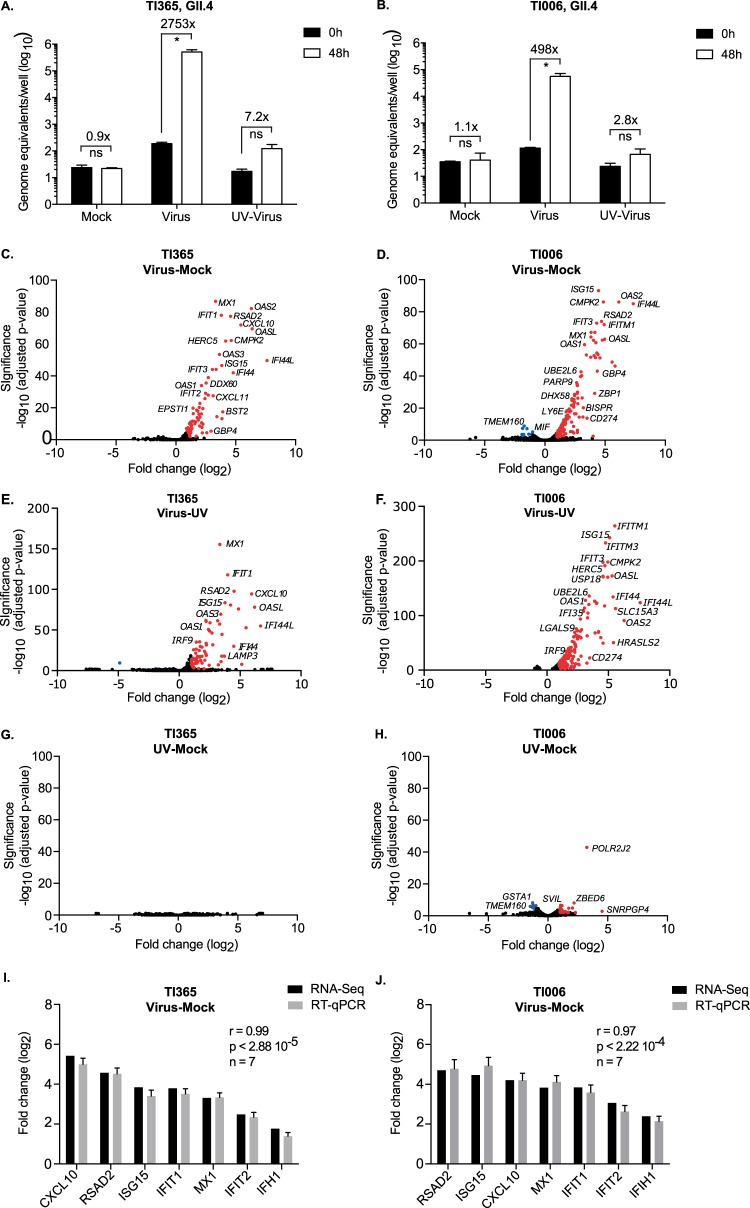
Norovirus infection of intestinal epithelial cells results in an interferon-induced transcriptomic response. (A and B) IECs derived from two terminal ileum organoid lines (TI365 [A] and TI006 [B]) were infected with an inactivatGII.4 HuNoV strain containing stool filtrate (Virus) or with the same stool filtrate after UV inactivation (UV-Virus) or mock infection (Mock), and the levels of viral RNA were quantified 48 h postinfection by RT-qPCR. Infections were performed in biological triplicate and quantified by RT-qPCR in technical duplicate. Error bars represent SEM. (C to F) Volcano plots representing differentially expressed genes from RNA-Seq analysis comparing gene expression levels in two different HuNoV-infected organoids (TI365 [C and E] and TI006 [D and F]) compared to mock treatment results (C and D) or the results seen with IEC infected with a UV-treated HuNoV inoculum (E and F). Panels G (TI365) and H (TI006) show volcano plots representing genes differentially expressed as identified by RNA-Seq analysis comparing IECs infected with UV-treated HuNoV inoculum to mock-infected cells. Significantly upregulated or downregulated genes (FDR < 0.01 and log_2_ fold change ≥ 1) are represented in red or blue, respectively. (I and J) Panels I (TI365) and J (TI006) show comparison of expression changes of selected genes following HuNoV infection measured by RNA-Seq and RT-qPCR. Error bars represent the standard deviations (SD) of results from one experiment performed in biological triplicate. The Pearson correlation coefficient (r), associated *P* value (p), and numbers of pairs analyzed (n) are indicated on each chart.

Robust infection of the cultures was evident from the increase in viral RNA levels over time, with 2,753-fold and 498-fold increases in HuNoV RNA levels seen in the TI365 and TI006 terminal ileum lines, respectively (Fig. 3A and B). As expected, only minimal increases in viral RNA levels were observed in IEC monolayers inoculated with UV-inactivated stool filtrate (Fig. 3A and B). To identify genes differentially regulated in response to productive HuNoV replication, pairwise gene comparisons were performed with mock-infected organoids and with IEC monolayers infected with UV-inactivated HuNoV or active HuNoV (Fig. 3C to H). Three biological repeats of each condition were analyzed by RNA-Seq as described in Materials and Methods. A total of 70 genes were found to be differentially regulated in GII.4 HuNoV IECs derived from organoid line TI365 compared to the mock-infected sample, with 69 increasing and 1 decreasing in their expression levels (Fig. 3C; see also Table S4). Results of comparisons of the active inoculum-infected TI365 samples to the samples infected with UV-inactivated inoculum showed a slight increase in the number of differentially regulated genes (76 in total, with 73 showing increased expression and 3 showing decreased expression) (Fig. 3E; see also Table S4). UV inactivation of the sample resulted in nearly complete ablation of the transcriptional response compared to the mock-infected cells, with very few of the results reaching statistical significance, confirming that the transcriptional signature was virus specific (Fig. 3G and H). In comparison, 162 genes were differentially regulated in GII.4 HuNoV-infected IECs derived from organoid line TI006 in comparison to mock-infected cells, with 9 genes showing decreases in expression and 153 showing increases (Fig. 3D; see also Table S4). The number of differentially regulated genes was reduced compared to number seen with the IECs infected with UV-inactivated inoculum (142 in total) (Fig. 3F; see also Table S4). Similarly to our observations obtained with GII.4 HuNoV infection of TI365, infection of TI1006 with the UV-inactivated inoculum resulted in a nearly complete loss of the transcriptional response.

In order to validate the results from the RNA-Seq analysis, we selected 7 differentially regulated genes and performed real-time quantitative PCR (RT-qPCR) on the same biological samples. We observed a strong correlation between the RT-qPCR and RNA-Seq results, confirming the accuracy of the expression data obtained by RNA-Seq (Fig. 3I and J).

**FIG 4:**
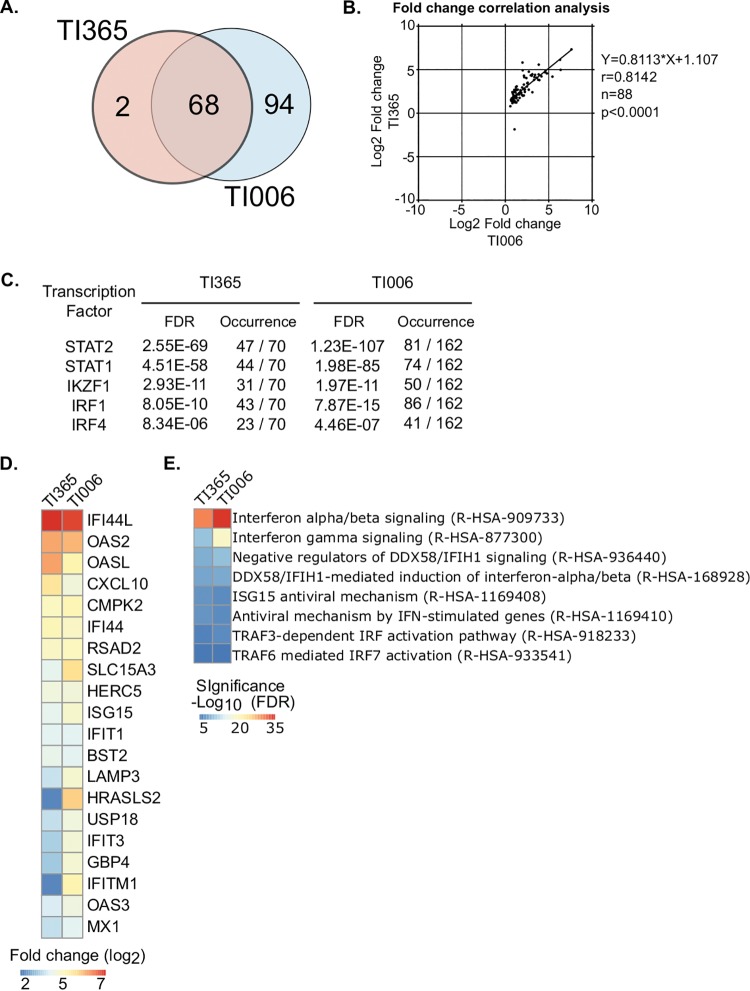
Human intestinal epithelial cells mount an interferon response to GII.4 human norovirus infection. (A) Venn diagram highlighting the common genes differentially expressed in HuNoV-infected cells generated from the two organoid lines, TI365 and TI006. (B) Fold change correlation analysis of statistically regulated genes common to two organoid lines. The equations representing the linear regression (Y), the Pearson correlation coefficient (r), the numbers of pairs analyzed (n), and the statistical significance (p) are indicated on the graph. (C) Transcription factor enrichment analysis from differentially expressed genes of IECs from two terminal ileum-derived organoid lines (TI365 and TI006). Enriched transcription factors, numbers of occurrences among significantly regulated genes, and statistical significance are indicated for each organoid infection. (D) Heat map showing the expression changes of the top 20 genes across two independent IEC infections. Genes are arranged by decreasing average enrichment for a false discovery rate (FDR) value lower than 0.01. (E) Heat map showing the most significant enriched Gene Ontology categories for biological processes inferred from significantly regulated genes across two independent organoid infections.

Results of comparisons of the genes found to be differentially regulated following HuNoV infection of the two organoid lines, TI365 and TI006, are plotted in the Venn diagram in Fig. 4A. We found that 70 genes were identified as being statistically differentially regulated following infection of the TI365 organoid line and 162 following infection of the TI006 line. Of these, 68 genes were common between the two lines and 94 were found to be solely regulated in the TI006 organoid. To further explore the similarity of these data sets, we performed a fold change (FC) correlation analysis and observed a high correlation between the gene expression levels measured in all of the organoid lines following HuNoV infection (Fig. 4B). The vast majority (73 of 94) of the genes solely regulated in TI006 organoid were classified as ISG according to the Interferome database (Table S4).

Transcription factor enrichment analysis unambiguously identified STAT1 and STAT2 binding sites as highly enriched in the promoter region of genes whose levels of expression were significantly regulated following infection of human intestinal organoids (Fig. 4C). This strongly suggested that the JAK-STAT signaling pathway is activated following HuNoV infection. In agreement, a heat map of the top 20 genes found to be upregulated across two independent IEC infections highlighted profound induction of type I interferon signaling in the transcriptomic response to HuNoV infection (Fig. 4D). In addition, a heat map showing the most significantly enriched gene ontology categories confirmed the involvement of interferon signaling in response to HuNoV infection (Fig. 4E).

Comparisons of the genes differentially expressed in response to HuNoV infection with data in the Interferome database revealed that 94% (*n* = 66) and 86% (*n* = 140) of these genes were categorized as ISGs in organoid lines TI365 and TI006, respectively. Overall, these results demonstrated that HuNoV infection is readily sensed by IECs and that the IFN-induced JAK/STAT signaling pathway is likely activated during HuNoV infection and/or active replication.

### Genetic modification of intestinal organoids to ablate interferon induction or interferon signaling enhances HuNoV replication in IECs.

**FIG 5:**
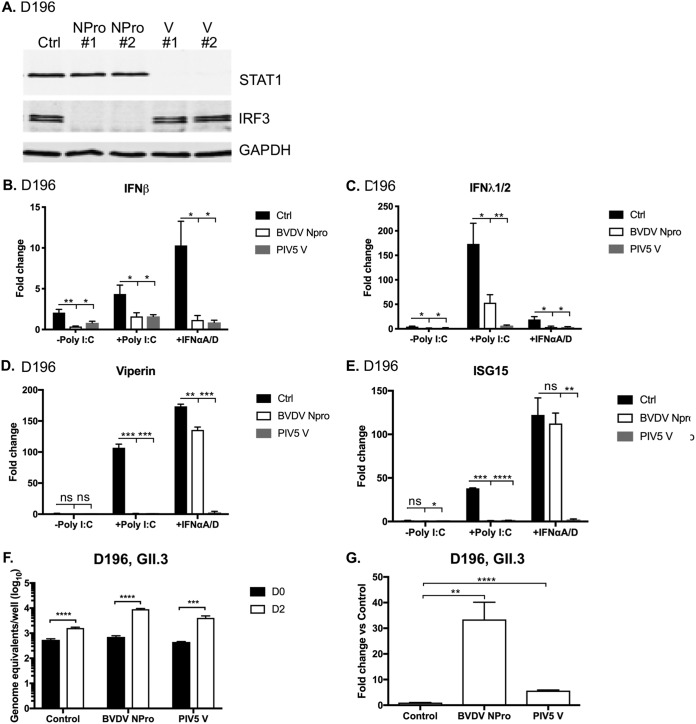
Genetically modified IFN-deficient organoids are more permissive for HuNoV replication. Duodenal intestinal organoids were modified by lentivirus-mediated transduction of the viral innate immune regulators BVDV NPro and PIV5 V proteins. (A) Two independent clones of transduced organoids were lysed, and the expression levels of STAT1, IRF3, and GAPDH (glyceraldehyde-3-phosphate dehydrogenase) were examined by Western blotting to confirm the functionality of the BVDV NPro or V protein in intestinal organoids. Ctrl, control. (B to E) To verify the inhibition of IFN production or IFN signaling, unmodified or modified intestinal epithelial cells were differentiated into monolayers and were transfected with poly(I·C) or treated with recombinant universal type I interferon (IFN-αA/D). The levels of IFN-β, IFN-λ1/2, viperin, and ISG15 were then quantitated by RT-qPCR. (B) IFN-β. (C) IFN-λ1/2. (D) Viperin. (E) ISG15. (F and G) Replication of HuNoV GII.3 was examined in IECs derived from IFN-deficient organoids (D196). The levels of viral RNA obtained 48 h postinfection (D2) were compared to those obtained 2 h postinfection (D0). The levels of viral RNA replication seen in modified organoids were expressed relative to that seen in the control unmodified organoid line and are expressed as genome equivalents (F) or fold change (G). All experiments were performed at least two independent times, and results are expressed as means ± SEM from duplicate samples analyzed in technical duplicate. Statistically significant values are represented as follows: *, *P* ≤ 0.05; **, *P* ≤ 0.01; ***, *P* ≤ 0.001; ****, *P* ≤ 0.0001.

To further examine the impact of IFN induction and the IFN signaling pathway on the restriction of HuNoV replication in IECs, we used lentiviral vectors to express viral innate immune antagonists to generate interferon-deficient intestinal organoid lines. Lentiviral vectors were used to drive constitutive expression of either bovine viral diarrhea virus (BVDV) NPro or parainfluenza virus type 5 (PIV5) V proteins, representing two well-characterized viral innate immune antagonists, in a duodenum-derived organoid line (D196). In brief, the BVDV NPro protein originates from a noncytopathic, persistent biotype of BVDV which effectively blocks IFN production by degrading interferon regulatory factor 3 (IRF3), thereby preventing the activation of the innate immune system (31, 32). The PIV5 V protein instead targets IFN production as well as antiviral signaling by targeting STAT1, MDA5, and LGP2 for proteasomal degradation (33–35). To confirm that the transduced proteins were functional, the expression levels of STAT1 and IRF3 were examined by Western blotting. While the STAT1 protein was present in the nontransduced control organoid and BVDV NPro-expressing organoid lines, no STAT1 protein was observed in PIV5 V-expressing cells (Fig. 5A). The IRF3 protein was not detected in BVDV NPro-expressing cells but was present in control and PIV5 V-transduced cells (Fig. 5A), confirming the functionality of NPro protein.

To verify the ability of NPro and V proteins to inhibit IFN induction and IFN signaling in the intestinal epithelium, IECs derived from stably transduced organoid lines were transfected with poly(I·C) or treated with recombinant universal type I IFN-αA/D (a hybrid between human IFN-α A and D). The levels of IFN-β and IFN-λ1 and of two representative ISGs, the viperin and ISG15 genes, were then quantified by RT-qPCR. Following poly(I·C) transfection, as expected, elevated levels of IFN-β, IFN-λ1, viperin, and ISG15 mRNAs were observed in control IECs transduced with the empty vector (Fig. 5B to E). In comparison, the IFN-β and IFN-λ1 mRNA induction levels were significantly lower in the BVDV NPro-expressing and PIV5 V-expressing cells, as were the induction levels of mRNAs for viperin and ISG15 (Fig. 5B to E). Following treatment with type I IFN, IECs expressing BVDV NPro protein or PIV5 V protein showed significantly reduced IFN-β and IFN-λ1 mRNA induction levels compared with the control cells (Fig. 5B and C). Expression of viperin and ISG15 mRNAs was not induced in IFN-treated PIV5 V-transduced IECs, confirming the impact of the V protein on IFN signaling (Fig. 5D and E). These data further confirm that the IECs were IFN competent and that the BVDV NPro and PIV5 V proteins could efficiently block IFN induction and signaling in IECs.

The ability of HuNoV to infect IECs from the transduced organoid line was then investigated. Infection of the nontransduced D196 line with a GII.3 HuNoV strain resulted in only modest levels of virus replication; an ∼6-fold increase in viral RNA was observed over 48 h in the control nontransduced line, suggesting that the process of replication of this isolate in the D196 line was inefficient (Fig. 5F). However, suppression of the innate response by expression of the NPro proteins or V proteins resulted in stimulation of GII.3 HuNoV replication in the D196 line; comparing the yield of viral RNA from IECs derived from the nontransduced D196 line to the yield obtained from the transduced IECs, we observed that the levels of GII.3 HuNoV replication were increased by ∼33-fold and ∼6-fold in the NPro protein-expressing and V protein-expressing D196-derived IECs, respectively (Fig. 5G). Furthermore, we found that HuNoV GII.3 infection of the transduced lines did not induce ISG15, confirming the functionality of the transduced innate immune antagonists in HuNoV-infected IECs (not shown). Similar results were obtained using a second transduced duodenal organoid line (results not shown). These results demonstrate that IECs produced from IFN-deficient organoids are more permissive for HuNoV and that the innate response limits HuNoV replication *in vitro*.

### Selective inhibition of JAK1/JAK2 enhances HuNoV replication in IECs.

**FIG 6:**
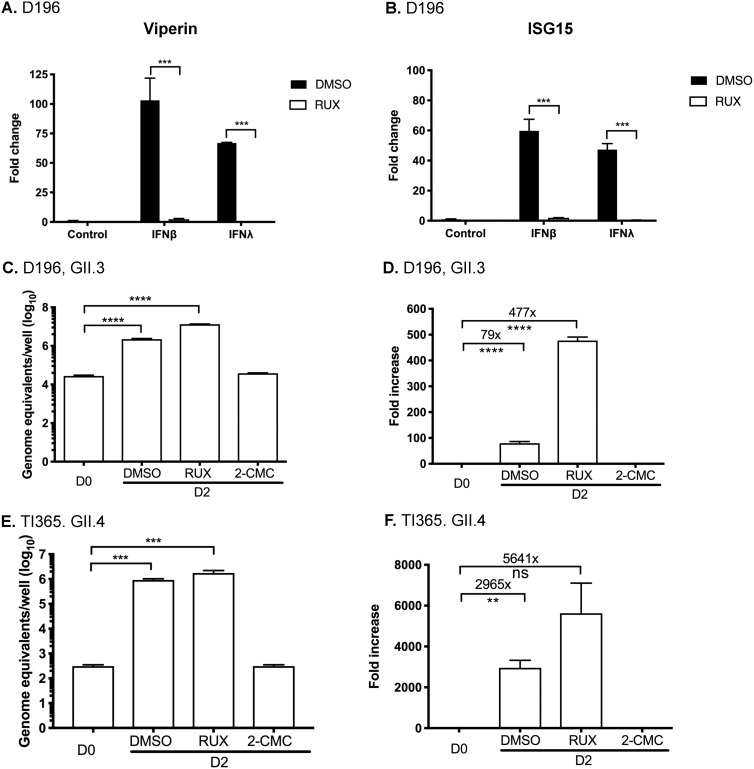
Inhibition of JAK1/JAK2 by ruxolitinib (RUX) enhances HuNoV replication in intestinal epithelial cells. (A and B) The ability of ruxolitinib (Rux) to inhibit type I and type III IFN signaling in the presence of viperin (A) or ISG15 (B) was examined following interferon (IFN-β or IFN-λ1/2) pretreatments of intestinal epithelial cells derived from duodenal intestinal organoids (D196). (C tot F) To investigate the impact the role of JAK signaling in the restriction of HuNoV replication, intestinal epithelial cells from D196 (C and D) and TI365 (E and F) were treated with DMSO, ruxolitinib (RUX), or 2-CMC (an inhibitor of HuNoV RNA-dependent RNA polymerase), and the impact of viral RNA synthesis was examined at 48 h postinoculation (D2) by RT-qPCR. Data are expressed as genome equivalents (C and E) or fold change (D and F). All experiments were performed at least three independent times, and results are expressed as means ± SEM from triplicate samples analyzed in technical duplicate. Statistically significant values are represented as follows: *, *P* ≤ 0.05; **, *P* ≤ 0.01; ***, *P* ≤ 0.001; ****, *P* ≤ 0.0001.

To further dissect the role of intestinal epithelial innate responses in the restriction of HuNoV infection, we investigated the effect of a specific Janus kinase 1 (JAK1)/JAK2 inhibitor on HuNoV replication in human IECs. Ruxolitinib (Rux) is a drug approved by the FDA for treatment for patients with dysregulated JAK signaling associated with myelofibrosis (36, 37) and for graft-versus-host disease (GvHD) (38). Rux has also been used to enhance growth of viruses that are sensitive to IFN (39). We first verified the ability of Rux to inhibit type I and type III IFN signaling following treatment of differentiated IEC monolayers derived from duodenal organoids with IFN-β or IFN-λ1/2. Rux pretreatment was able to efficiently block the induction of viperin and ISG15 mRNAs following treatment with IFN-β or IFN-λ1/2 (Fig. 6A and B). We then examined the effect of Rux on HuNoV replication in IECs derived from the proximal duodenum and terminal ileum (Fig. 6C to F). Differentiated IEC monolayers were inoculated with either GII.3 HuNoV-positive or GII.4 HuNoV-positive stool filtrates. The inoculum was removed after 2 h, and the cells were washed and maintained in bile acid (GCDCA)-containing media supplemented with dimethyl sulfoxide (DMSO), Rux, or 2-*C*-methylcytidine (2-CMC). The 2-CMC RNA polymerase inhibitor was included as a control as a well-characterized inhibitor of HuNoV replication in replicon-containing cells (40).

The impact of Rux treatment on HuNoV replication was assessed at 2 days pi by qRT-PCR, and the results confirmed that inhibition of JAK resulted in stimulation of HuNoV replication. In the absence of Rux, we observed ∼79-fold and ∼2,965-fold increases in HuNoV GII.3 and GII.4 viral RNA levels, respectively, in IECs derived from duodenal and terminal ileum organoids, respectively (Fig. 6C to F). The inclusion of Rux in cultures following inoculation resulted in a significant improvement in HuNoV replication in all cases; GII.3 replication was increased ∼477-fold and GII.4 replication ∼5,641-fold over a 48-h period (Fig. 6C to F). In all cases, the addition of 2-CMC inhibited HuNoV replication, resulting in levels of viral RNA that were nearly identical to those observed at D0 pi (Fig. 6C to F). Rux stimulated the replication of a number of HuNoV isolates in IECs derived from a variety of duodenum and terminal ileum organoid lines (see Fig. S1 in the supplemental material). These data confirm that activation of JAK1/JAK2 inhibits HuNoV replication and that pharmacological inhibition of this pathway increased HuNoV replication in culture.

10.1128/mBio.00215-20.1 FIG S1 Ruxolitinib stimulates the replication of GII.4 in intestinal epithelial cells from mucosa-derived intestinal epithelial organoids (IEOs). Intestinal organoids were generated from biopsy specimens derived from the duodenum of four patients (lines designated D353, D419, D421, and D428) or from the terminal ileum of a single patient (TI006). Monolayers of intestinal epithelial cells (IECs) were generated as described in the text and infected with either GII.3 or GII.4 human norovirus stool filtrate. The levels of viral RNA were quantified at day 0 (D0) or 48 h postinfection (D2) by RT-qPCR and plotted as either genome equivalents per well (left column) or fold increase in viral RNA over 48 h (right column). The impact of the inclusion of the JAK inhibitor (RUX) or the RNA polymerase inhibitor (2-CMC) on viral replication was examined by the addition of RUX or 2-CMC following the inoculation phase of the infection. All experiments were performed independently at least three times, and results are expressed as means ± SEM from triplicate samples analyzed in technical duplicate. Significant values are represented as follows: *, *P* ≤ 0.05; **, *P* ≤ 0.01; ***, *P* ≤ 0.001; ****, *P* ≤ 0.0001. Download FIG S1, PDF file, 0.3 MB. Copyright © 2020 Hosmillo et al. 2020 Hosmillo et al. This content is distributed under the terms of the Creative Commons Attribution 4.0 International license.

### Inhibition of RNA polymerase II-dependent transcription increases HuNoV replication in IECs.

To further assess the impact of *de novo* transcriptional responses on the restriction of HuNoV replication in IECs, we examined the impact of triptolide (TPL), a compound extracted from a traditional Chinese medicinal plant (Tripterygium wilfordii Hook F), on HuNoV replication in culture. TPL has potent immunosuppressant and anti-inflammatory activities, exhibiting broad pharmacological effects against inflammation, fibrosis, cancer, viral infection, oxidative stress, and osteoporosis (41, 42). TPL is known to have both antiproliferative and proapoptotic effects on a range of cancers (43, 44) and was previously reported to modulate the activity of many genes, including those involved in apoptosis and NF-κB-mediated responses (42, 45). RNA polymerase II was recently shown to be selectively targeted by TPL, although the mechanism by which TPL inhibits RNA polymerase II activity is yet to be fully elucidated (46). One of the known effects of TPL is the rapid depletion of short-lived RNAs, including those associated with transcription factors, cell cycle regulators, and oncogenes (41, 46). Recent work has also confirmed that TPL treatment inhibits the innate response and stimulates vesicular stomatitis virus-induced oncolysis (47).

**FIG 7:**
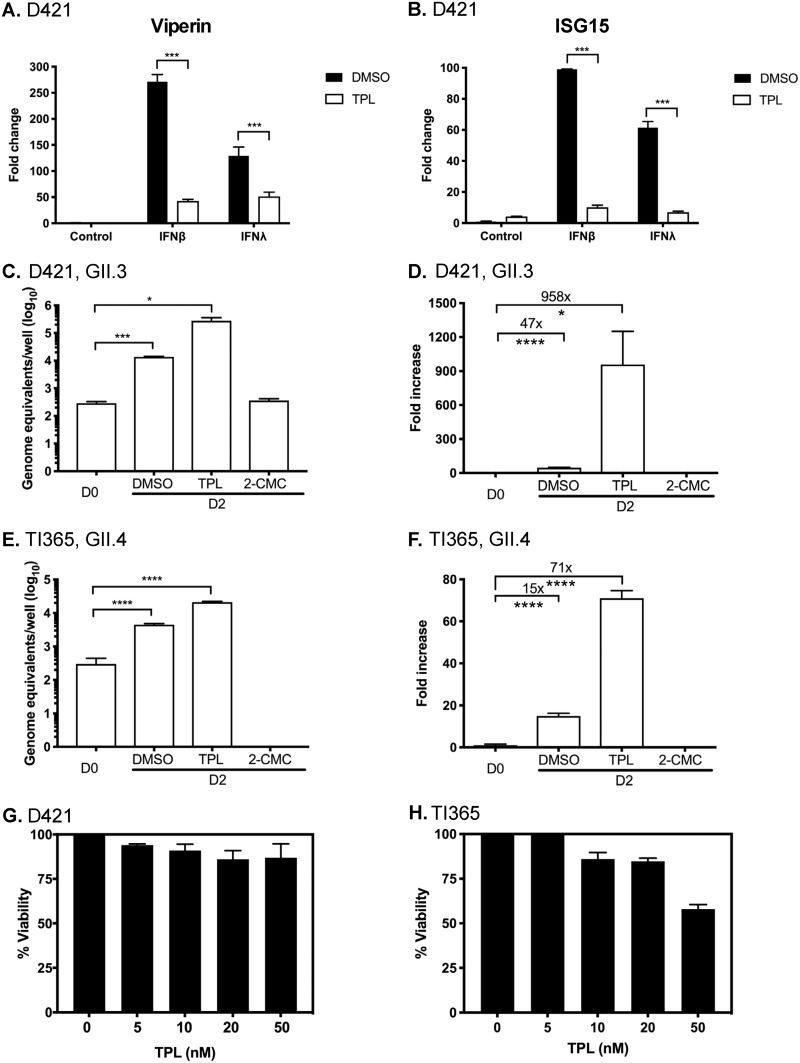
Inhibition of cellular transcription by triptolide (TPL), an RNA polymerase II (RNAPII) inhibitor, enhances HuNoV replication in intestinal epithelial cells. (A and B) The ability of triptolide to inhibit type I and type III IFN signaling was examined following interferon pretreatment of intestinal epithelial cells derived from duodenal intestinal organoids (D421). (A) Viperin. (B) ISG15. (C to F) To investigate the impact of restriction of transcription factors in HuNoV GII.3 and GII.4 replication, intestinal epithelial cells derived from duodenum (D421) (C and D) and ileum (TI365) (E and F) were treated with DMSO, triptolide (TPL), or 2-CMC (an inhibitor of HuNoV polymerase), and the impact of viral RNA synthesis was examined at 48 h postinoculation (D2) by RT-qPCR. Data are expressed as genome equivalents (C and E) or fold change (D and F). (G and H) To ensure that concentration of TPL used in infections is noncytotoxic, percentages of viable cells were monitored in D421 (G) and TI365 (H) monolayers following 0, 5, 10, 20, and 50 nM treatments. All experiments were performed at least three independent times, and results are expressed as means ± SEM from triplicate samples analyzed in technical duplicate. Significant values are represented as follows: *, *P* ≤ 0.05; **, *P* ≤ 0.01; ***, *P* ≤ 0.001; ****, *P* ≤ 0.0001.

To examine the effect of inhibition of transcription by TPL on the capacity of IECs to respond to IFN, the levels of viperin and ISG15 were assessed following treatment of cells with IFN-β or IFN-λ. The mRNA levels of viperin was increased by 100-fold to 250-fold after treatment with IFN-β1 or IFN-λ1/2 in DMSO-treated control IEC monolayers, but these increases were almost completely suppressed by the inclusion of TPL (Fig. 7A). A similar observation was made for ISG15, in that the inclusion of TPL potently inhibited the induction by interferon (Fig. 7B). These data confirm that the use of TPL at concentrations that do not affect overall cell viability is effective at suppressing the innate response in IECs.

To examine the effect of TPL on HuNoV replication, differentiated monolayers generated from proximal duodenum and terminal ileum were inoculated with either GII.3 HuNoV-positive or GII.4 HuNoV-positive stool filtrates. After 2 h, the inoculum was removed and the cells were washed and maintained in GCDCA-containing differentiation media with DMSO, TPL, or 2-*C*-methylcytidine (2-CMC) as a control. The addition of TPL resulted in enhanced HuNoV replication of GII.3 and GII.4 HuNoV strains by the use of the concentration (10 nM) of TPL that demonstrated the presence of at least 86% viable cells (Fig. 7G and H). These observations were consistent in IECs derived from both the duodenum (Fig. 7C and D) and terminal ileum (Fig. 7E and F). As expected, HuNoV replication was potently inhibited in the presence of 2-CMC (Fig. 7C to F). Enhancement of viral replication was also observed in other duodenum and terminal ileum organoid lines (Fig. S2). These results further confirm that inhibition of IFN-induced transcription increases HuNoV infection.

10.1128/mBio.00215-20.2 FIG S2 Triptolide stimulates the replication of GII.3 or GII.4 in intestinal epithelial cells from mucosa-derived intestinal epithelial organoids (IEOs). Intestinal organoids were generated from biopsy specimens derived from the duodenum (line D428) or the terminal ileum of a single patient (TI006). Intestinal epithelial cell monolayers (IEC) were generated as described in the text and infected with a GII.4 human norovirus stool filtrate. The levels of viral RNA were quantified at day 0 (D0) or at 48 h postinfection (D2) by RT-qPCR and plotted as either genome equivalents per well (left column) or fold increase in viral RNA over 48 h (right column). The impact of the inclusion of the RNA polymerase II inhibitor (TPL) or the viral RNA polymerase inhibitor (2-CMC) on viral replication was examined by the addition of TPL or 2-CMC following the inoculation phase of the infection. All experiments were performed at least three independent times, and results are expressed as means ± SEM from triplicate samples analyzed in technical duplicate. Significant values are represented as follows: *, *P* ≤ 0.05; **, *P* ≤ 0.01; ***, *P* ≤ 0.001; ****, *P* ≤ 0.0001. Download FIG S2, PDF file, 0.1 MB. Copyright © 2020 Hosmillo et al. 2020 Hosmillo et al. This content is distributed under the terms of the Creative Commons Attribution 4.0 International license.

## DISCUSSION

Efficient cultivation of HuNoV has remained a challenge since the initial identification of the prototype norovirus, Norwalk virus, in 1972 (48). Norovirus infection of the natural host species is very efficient, typically requiring <20 virus particles to produce a robust infection whereby >10^8^ viral RNA copies are shed per gram of stool within 24 h (49, 50). Even in heterologous hosts (e.g., pigs), the HuNoV infectious dose has been estimated to be ∼2 × 10^3^ viral RNA copies (51). Despite this, and despite enormous efforts, the lack of ability to culture HuNoV efficiently has represented a significant bottleneck in the study of HuNoV biology (48). Therefore, the ability to culture HuNoV has the potential to transform our understanding of many aspects of the norovirus life cycle, to greatly enhance the capacity to develop therapeutics, and to allow the characterization of authentic viral neutralization titers following vaccination rather than the use of the current surrogate gold standard method (21, 23). The net result of >40 years of research is the establishment of two culture systems for HuNoV that use patient stool samples as the inoculum. The first such system relies on the replication of HuNoV within immortalized B cells and requires the presence of enteric bacteria or soluble histo-blood group antigen (HBGA)-like molecules from their surface (21, 22). While we have been able to reproduce the culture of HuNoV in immortalized and primary B cells with various degrees of success (data not shown), we note that attempts in other laboratories have not universally been successful (21).

The recently developed HuNoV culture system IECs derived from intestinal organoids (23), while experimentally challenging, has been used in a number of subsequent studies to examine the impact of disinfectants (52) and of monoclonal antibodies (53, 54). This study set out to use an organoid-based system to assess the cellular pathways that restrict HuNoV replication and to further refine the experimental conditions that allow optimal growth of HuNoV in culture. We found that HuNoV infection induced a robust innate response in IECs, in contrast to previous studies that used transfection of purified HuNoV RNA into immortalized cells and which concluded that the interferon response is unlikely to play a role (28). While the conclusions drawn in that previous study may be valid, it is likely that the inefficient replication seen using transfected RNA, where less than 0.1% of transfected cells contained active replicating viral RNA, resulted in a reduction of the sensitivity of the experimental system. The results may have been further confounded by the presence of unknown mutations that affected the robustness of the sensing pathways within immortalized cells. Those previous observations also contrast with our own previous findings that suggested that the ability of cells to respond to exogenous interferon negatively impacts HuNoV replication (55, 56). That conclusion was based on the finding that the IFN-λ receptor is epigenetically suppressed in an immortalized intestinal cell line which efficiently replicates a HuNoV GI replicon and that genetic ablation of IFN-λ receptor expression enhances HuNoV replication in immortalized cells (56). We also recently described the generation of a robust culture system in zebrafish larvae in which we also observed MX and RSAD2 (viperin) induction (30). Furthermore, it is well established that the interferon response is key to the control of MNV infection, as mice lacking a competent innate response often succumb to lethal systemic MNV infections (57–59), demonstrating that the innate response is key to the restriction of norovirus infection to intestinal tissues in the mouse model (60). The development of the HuNoV organoid culture system provides the first opportunity to assess the impact of HuNoV infection on IECs, the first port of entry into the natural host.

Here, we have seen that HuNoV infection of IECs induces an IFN-like transcriptional response by examining the replication of single HuNoV GII.4 isolates in IECs derived from two independent terminal ileum organoid lines from two different donors (Fig. 3). We chose the terminal ileum-derived organoids as our source of IECs because our data to date would suggest that GII.4 HuNoV replicated more efficiently in IECs derived from this gut segment whereas the GII.3 isolate replicated more efficiently in duodenal lines (Fig. 1D). Whether this difference was organoid line or viral strain specific or suggests differing tropisms is unknown; however, this observation was consistent across several different duodenal and ileal organoid lines (data not shown).

Under the conditions used in the current study, the overall numbers of genes that were found to have altered more than 2-fold in response to infection were relatively modest at 70 and 162 for TI365 and TI1006, respectively. We found that the transcriptional responses induced in each organoid line were highly comparable, with a substantial overlap in the induced genes (Fig. 4). The use of UV-inactivated inoculum allowed us to control for any nonspecific effects of the other components of the filtered stool sample. Given the heterogeneity of any given stool sample, this inclusion was essential to ensuring that the observations were robust and represented alterations due to sensing of active viral replication intermediates. The rather modest number of induced genes likely reflects the heterogenous nature of the IEC cultures and that not all cells in any given monolayer are permissive to infection. We estimate that ∼30% of the cells were typically infected under the conditions used for the gene expression analysis, which is similar to previous reports (23). The inclusion of Rux or TPL increased the overall number of infected cells in any given culture to ∼50%, but even under the modified conditions, we have been unable to obtain higher levels of infection (data not shown). We hypothesize that obtaining higher levels of infection would likely require more-uniform cultures, consisting primarily of enterocytes, the target cell for HuNoV (23).

The mechanism by which HuNoV is sensed by the infected cells is not currently known; however, data from MNV suggest a clear role for Mda5-mediated sensing in the restriction of norovirus replication both in cell culture and *in vivo* (61). The sensing of MNV RNA occurs in a process that requires the HOIL1 component of the linear ubiquitin chain assembly complex (LUBAC) complex (62). Other components of the RNA sensing pathways, including mitochondrial antiviral-signaling protein (MAVS), IRF3, and IRF7, have been implicated in the innate response to MNV (61, 62), but the role that they play in sensing of HuNoV RNA is unknown. In addition to targeting STAT1 for degradation (63), the PIV5 V protein is known to also inhibit the activity of Mda5 (35). While not directly assessed, it is therefore likely that the stimulation of HuNoV replication in the presence of the PIV5 V protein represents the combined effects of both of these activities. Further studies using gene-edited organoid lines will be required to better define the relative contributions of the components in the sensing of HuNoV.

The gene most highly induced in response to HuNoV infection in both organoid lines was the IFI44L gene, encoding a novel tumor suppressor (64) previously show to have modest antiviral activity against hepatitis C virus (HCV) (65) and respiratory syncytial virus (RSV) (66, 67). IFI44L was also potently upregulated in IECs infected with human rotavirus (HRV) (68). Surprisingly, despite inducing a potent interferon response in IECs, HRV is not restricted by the endogenously produced IFN (68), an effect that has been hypothesized to be due to viral regulatory mechanisms that suppress the downstream activities of the induced genes. A number of the genes induced in response to HuNoV infection of IECs have previously been shown to have antiviral activity against noroviruses. Guanylate binding protein 4 (GBP4) and GBP1 were both induced following GII.4 infection of both organoid lines (Fig. 3). The GBPs are interferon-induced proteins that are targeted to membranes of vacuoles that contain intracellular fungi or bacterial pathogens (69, 70), where their presence frequently results in disruption of the pathogen-containing vacuoles (70). GBPs are targeted to the MNV replication complex in an interferon-dependent manner that requires components of the autophagy pathway and exert their antiviral activity via an unknown mechanism (71). GBP2 was also identified as a norovirus restriction factor in a CRISPR-based activation screen where it was found to have potent antiviral activity against two strains of MNV (72). Further studies will be required to determine if GBPs have similar antiviral effects during HuNoV infection.

The IFIT proteins IFIT1 to IFIT3 were also significantly induced in response to HuNoV infection of IECs (Fig. 3; see also Table S1 in the supplemental material). The IFITs are members of a family of interferon-stimulated RNA binding proteins that, at least in humans, are thought to inhibit the translation of foreign RNAs by binding to 5′ termini and preventing translation initiation (73, 74). We have recently shown that translation of norovirus VPg-linked RNA genomes in the context of norovirus infection is not sensitive to IFIT1-mediated restriction (75), most likely due to the mechanism associated with the novel VPg-dependent manner by which norovirus RNA is translated (75). However, we did observe that IFIT1 in some way enhanced the IFN-mediated suppression of norovirus replication through an as-yet-undefined mechanism (75).

The development of the B-cell and organoid culture system has opened up the opportunity to dissect the molecular mechanisms of norovirus genome replication and to better understand host responses to infection. Others have observed that HuNoV replication in organoid-derived IECs is highly variable (76), which agrees with our own experience during the course of the current study, as we observed significant levels of week-to-week variations in infectious yield from the same organoid lines for any single strain of HuNoV (data not shown). While several factors likely contribute to the variability, compiling our data over a period of time would suggest a general correlation with differentiation status and with the passage number of the organoids (data not shown). As a result, high-passage-number organoid lines, such as transduced lines, appear to have lower levels of differentiation, resulting in reduced viral yields. We have also observed, as have others, that not all HuNoV strains appear to replicate efficiently in IECs derived from any single organoid line, which likely reflects the natural biology of HuNoV, as the individual levels of susceptibility differ within any given population (76). What factors contribute to the relative levels of permissiveness of any given organoid line to an isolate of HuNoV remains to be determined, but it is clear that FUT2 function appears essential for most HuNoV isolates as FUT2-negative lines were not permissive to the strains of viruses tested here (data not shown) (23, 76). It is also possible that the strains differ in the degrees to which they induce and are sensitive to the interferon response, as is common for other positive-sense RNA viruses. Our data would seem to suggest that, irrespective of this, replication of all isolates examined appeared to be improved by treatment of cultures with Rux or TPL (Fig. 6 and 7; see also Fig. S2 in the supplemental material) (additional data not shown). An issue that remains to be fully addressed is that of whether the increased replication observed in the presence of Rux or TPL was due an increased number of permissive cells in any given differentiated culture or simply to enhanced RNA synthesis in each infected cell. The former might arise due to an increased number of productive infections. Addressing this issue is technically challenging due to the nature of the organoid culture system, but an initial analysis indicates that an increased number of cells expressing detectable levels of viral antigen were detected in Rux-treated intestinal epithelial cells.

To our knowledge, our study represents the first demonstration that genetic modification of human intestinal organoids can improve viral replication. The expression of BVDV NPro and PIV5 V proteins in cells has been widely used as a way to enhance virus replication in immortalized cells via the inactivation of aspects of the innate response (18, 77, 78). While the genetically modified organoids were found to have enhanced HuNoV replication up to 30-fold in comparison to unmodified organoids, we found that the results differed between the organoid lines examined (data not shown). Surprisingly, we found that the process of differentiation resulted in a significant increase in the basal levels of a number of ISGs (data not shown). Therefore, the reason for variation in the enhancement is unknown but may be related to the ability of any given organoid line to respond effectively and produce a rapid and effective innate response. While the ability to readily generate gene-edited human intestinal organoids has been developed, the method is still very much in its infancy (79); therefore, the ability to overexpress viral innate immune antagonists represents a more rapid way of generating intestinal organoids with specific defects in innate immune pathways. However, simple inclusion of TPL or Rux appears to phenocopy the effect of overexpression of either NPro or V protein and can be readily applied to any organoid line. This low-cost modification to culture conditions enhances the utility of the experimental system by improving the robustness of the replication.

The use of pharmacological inhibitors for the stimulation of viral infection has been described in many studies of immortalized cell lines (39, 47, 80) and more recently for viral infection of intestinal organoids (81). The mechanism of action of Rux is well defined, as it specifically targets the JAK kinases (36). In contrast, the mechanism of action of TPL is less well defined but recent data suggest a direct mode of action in RNA polymerase II-mediated transcription (46). TPL has previously been shown to stimulate the replication of VSV by the inhibition of the interferon induced transcriptional responses (47). While TPL is not clinically used due to problems with water solubility, a water-soluble prodrug, minnelide, has been the subject of trials investigating its efficacy as an anticancer treatment for a number of cancers, including pancreatic cancer (82).

Norovirus infection has now been widely accepted to be a significant cause of morbidity and mortality in immunocompromised patients (13). In such cases, patients on immunosuppressive therapy following organ or stem cell replacement therapies, or those undergoing treatment for cancer, often suffer from infection lasting months to years (13, 83). Such infections have a profound impact on the overall health of the affected patient, resulting in significant weight loss and a requirement for enhanced nutritional support (84). Ruxolitinib (Jakavi) is approved for the treatment of a range of diseases, including splenomegaly, in patients with myelofibrosis and has been shown to be effective in the treatment of both the chronic and acute forms of the disease (38, 85). Our data might suggest that the sustained administration of Rux in patients where chronic norovirus infection has been detected may exacerbate the disease. We note, however, that during a study examining the effect of Rux on NK cell function in patients with STAT1 gain-of-function mutations, a single patient with chronic norovirus infection appeared to clear the infection following Rux treatment (86). The impact of Rux treatment on viral loads, and whether clearance was spontaneous or due to improved NK cell function, was not reported.

In summary, we have demonstrated that HuNoV replication in IECs is restricted by the interferon response and that modulation of this response through either the genetic manipulation of intestinal organoids or the inclusion of pharmacological inhibitors enhances HuNoV replication. Overall, this report provides new insights into the cellular pathways and processes that control the replication of HuNoV and provides improved conditions for the culture of HuNoV, enhancing the robustness of the HuNoV organoid culture system.

## MATERIALS AND METHODS

### Stool samples.

Stool specimens were anonymized with written consent from patients at Addenbrooke’s Hospital, Cambridge, United Kingdom, who tested positive of HuNoV infection. Stool samples were diluted 1:10 (wt/vol) with phosphate-buffered saline (PBS) and processed as described previously (23). Briefly, 10% stool suspensions were subjected to vigorous vortex mixing for 1 min and sonicated three times for 1 min each time (50/60 Hz, 80 W). Homogenous fecal suspensions were centrifuged at 1,500 × *g* for 10 min at 4°C. The supernatants were serially passed through 5-μm-, 1.2-μm, 0.8-μm, 0.45-μm-, and 0.22-μm-pore-size filters (Millex-GV syringe filter units). Stool filtrates were divided into aliquots and stored at –80°C until use.

### Human intestinal organoids.

Following ethical approval (REC-12/EE/0482) and informed consent, biopsy specimens were collected from the proximal duodenum (D) or terminal ileum (TI) of patients undergoing routine endoscopy. All patients included had macroscopically and histologically normal mucosa. The biopsy samples were processed immediately, and intestinal epithelial organoids were generated from isolated crypts following an established protocol as described previously (23, 27, 87). Intestinal organoids were grown in proliferation media (see Table S1 in the supplemental material) as described previously (27). Organoids were typically grown for 7 to 9 days prior to passage at ratios of 1:2 to 1:3.

Following the establishment of organoid cultures, differentiated IEC monolayers were generated on collagen-coated wells in differentiation media (Table S1) as described previously (23). Following 5 days of differentiation, confluent monolayers of differentiated IECs were infected. Differentiation was assessed by RT-qPCR at various time postinfection by assessing the levels of the stem cell marker LGR5, a mature enterocyte marker alkaline phosphatase (ALP), and epithelial cell marker villin (VIL). Data were normalized to the hypoxanthine phosphoribosyltransferase 1 (HPRT1) housekeeping gene.

### Cell lines and reagents.

L-WNT 3A-expressing cell lines were used to produce WNT-conditioned media as a component of the proliferation media and were propagated in a mixture containing low-glucose Dulbecco’s modified Eagle’s medium (DMEM) (Life Technologies), 10% fetal calf serum (FCS), 1% penicillin-streptomycin (P/S), and Zeocin (125 μg/ml) at 37°C with 5% CO_2_. WNT-conditioned medium was collected from cells grown in the absence of Zeocin. The activity of WNT3a in conditioned medium was assessed using a luciferase reporter assay reliant on a Wnt3A-responsive promoter (HEK 293 STF; ATCC CRL-3249).

293T-RSPO-V5 cells were used to produce R-Spondin 1 (RSPO1)-conditioned media. The 293T-RSPO-V5 cells were propagated in a mixture containing DMEM (Life Technologies), 10% fetal calf serum (FCS), 1% penicillin-streptomycin (P/S), and Zeocin (300 μg/ml) at 37°C with 5% CO_2_. RSPO1-conditioned media were collected from passages of cells in conditioned media containing DMEM/F12 (Life Technologies),1% penicillin-streptomycin (P/S), 10 mM HEPES (Life Technologies), and 1× GlutaMAX (Life Technologies).

Components of the proliferation and differentiation media are described in Table S1. The commercial sources of the interferons (IFN-αA/D, Sigma; IFN-β1, IFN-λ1, and IFN-λ2, Peprotech) and of IFN inhibitors ruxolitinib (InvivoGen) and triptolide (InvivoGen) are detailed in Table S2.

10.1128/mBio.00215-20.4 TABLE S2 Supplier details of the interferons and inhibitors used in this study. Download Table S2, XLSX file, 0.01 MB. Copyright © 2020 Hosmillo et al. 2020 Hosmillo et al. This content is distributed under the terms of the Creative Commons Attribution 4.0 International license.

### Lentivirus vector particle production and transduction.

Lentivirus transfer vectors encoding the BVDV NPro and PIV5 V proteins were a gift from Steve Goodbourn (St. George’s Hospital, University of London). The transfer vectors were used to generate vesicular stomatitis virus G protein-pseudotyped lentiviral particles by transfection of 293T cells with psPAX2 and pMD2.G helper plasmids. Human IEOs were then transduced with lentivirus-containing supernatants following published protocols (18). In brief, organoids growing in proliferation media were collected at D5, mechanically broken by pipetting, and centrifuged at 900 × *g* for 5 min. The pellet of organoids was incubated in TrypLE express dissociation medium (Gibco) for 5 min at 37°C and dissociated by repeated pipetting to achieve fragments consisting of 5 to 10 cells. Dissociation was terminated with differentiation media, and the fragments were centrifuged at 900 × *g* for 5 min. The organoid fragments were transferred to 48-well plates and subjected to spinoculation with lentiviruses prepared as described above in proliferation media supplemented with Rho-associated, coiled-coil-containing protein kinase (ROCK) inhibitor (10 μM) and Polybrene (8 μg/ml) for 1 h at 32°C at 600 × *g*. The plates were then incubated further for 6 h at 37°C with 5% CO_2_. Following transduction, transduction media and the organoid fragments were centrifuged at 900 × *g* for 5 min. The transduced cell pellets were then resuspended in Matrigel, and the organoid fragments were seeded into proliferation media and ROCK inhibitor (10 μM). The transduced organoids were then selected after 48 h with puromycin (2 μg/ml). The organoid clones were selected by limiting dilution and subsequent functional analysis.

### HuNoV infection.

Differentiated monolayers in 48-well plates were infected in biological duplicate or triplicate as described in the text. HuNoV stool filtrates containing ∼1 × 10^6^ viral RNA copies, determined by RT-qPCR, were added to each well and incubated 37°C for 2 h, prior to being washed twice with serum-free media, and were then overlaid with 250 μl of differentiation media containing 200 μM glycochenodeoxycholic acid (GCDCA; Sigma). Where required, wells were supplemented with DMSO, IFN, or pharmacological inhibitors as described in the text. Samples were typically harvested at 48 h postinfection for analysis.

Inactivated HuNoV-containing stool filtrates were prepared by placing stool filtrate into multiple 24-well plates to reach a fluid depth of 10 mm and exposing the filtrate to 4,000 mJ from a UV source for 12 min at 4°C. Loss of viral infectivity was confirmed by infection of monolayers and by comparison of the viral titers observed after 48 h postinfection with that obtained using the well-characterized RNA polymerase inhibitor 2-CMC (40).

### qRT-PCR and qPCR analysis.

Gene-specific primers and probes against the cellular mRNAs HPRT, LGR5, ALP, and VIL (Thermo Fisher Scientific) were used to evaluate differentiation by RT-qPCR. Samples were analyzed by technical duplicate qPCRs and the results averaged.

HuNoV GII-specific primers that had been reported previously (22) were used in TaqMan-based qRT-PCR assay to detect HuNoV replication in organoid cultures (Table S3). The levels of HuNoV mRNA were determined based on absolute quantitation against a standard curve generated using *in vitro*-transcribed RNA from a full-length cDNA clone of a GII.4 HuNoV. Each individual biological sample was analyzed by qRT-PCR in technical duplicate alongside additional no-template negative controls. Data were collected using a ViiA 7 real-time PCR system (Applied Biosystems).

10.1128/mBio.00215-20.5 TABLE S3 Primer sequences used for RT-qPCR and qPCR analyses performed in this study. Download Table S3, XLSX file, 0.01 MB. Copyright © 2020 Hosmillo et al. 2020 Hosmillo et al. This content is distributed under the terms of the Creative Commons Attribution 4.0 International license.

10.1128/mBio.00215-20.6 TABLE S4 Summary of the results of differential gene expression analysis of GII.4-infected intestinal epithelial cells. RNA-Seq analysis was performed as described in the text. The data shown represent averages obtained from the two biological repeats. Differential gene expression analysis was performed by comparing the data obtained from infected IECs to those obtained from mock-infected IECs (tabs A and C) or by comparing the data obtained from IECs infected with active inoculum to those obtained from IECs infected with UV-inactivated inoculum. Download Table S4, XLSX file, 0.5 MB. Copyright © 2020 Hosmillo et al. 2020 Hosmillo et al. This content is distributed under the terms of the Creative Commons Attribution 4.0 International license.

### RNA library preparation and sequencing.

Total cellular RNA was extracted from IECs using TRIzol (Invitrogen), and genomic DNA was removed by DNase I digestion (Turbo DNA-free kit; Ambion catalog no. AM1907). RNA integrity was assessed via the use of an Agilent 2200 TapeStation system and RNA ScreenTape reagents (Agilent Technologies catalog number 5067-5576/77). Libraries were prepared for sequencing by Cambridge Genomics Services by the use of 300 ng of total RNA and a TruSeq Stranded mRNA kit (Illumina Technologies catalog number 20020595). Libraries were quantified by qPCR and were pooled and sequenced with 75-bp single reads to a depth ranging from 13 to 55 million reads per sample using a Illumina NextSeq 500 system and a high-output 75-cycle kit (Illumina catalog number FC-404-2005).

### Data analysis.

Raw reads were inspected with FastQC. Adapters and low-quality sequences were removed by the use of Trimmomatic version 0.33 and the following parameters: ILLUMINACLIP:TruSeq3-SE:2:30:10 LEADING:3 TRAILING:3 SLIDIN GWINDOW:4:15 MINLEN:36. Quantification of transcript levels was performed using kallisto software (88) for each sample against the human transcriptome GRCh38.p12 (Ensembl release v92; accessed 22 March 2018), and genes with an average of less than 1 transcript per million under both control and virus infection conditions were excluded from downstream analysis. Read counts were normalized using the trimmed mean of M-values normalization method (89), and log_2_ counts per million (CPM) were obtained using the calcNormFactors and voom (90) functions of the edgeR (89) and limma (91) packages, respectively. Student’s *t* tests were then applied for each transcript. Finally, *P* value*s* were adjusted for false discoveries occurring due to simultaneous testing of hypotheses by applying the Benjamini-Hochberg procedure (false-discovery rate [FDR]) (92). Transcripts with a FDR value lower than 0.01 and log2FC value greater than 1 (FC = 2) were considered differentially expressed. A heat map of gene expression for significantly expressed genes across comparisons was generated using R package pheatmap version 1.0.10. Levels of expression change are represented in the figures by a color gradient ranging from blue (low increase in gene expression) to red (high increase in gene expression). Gene ontology term and Reactome pathway enrichment analyses were performed with clusterProfiler R package version 3.8.1 (93) and represented using the R package pheatmap as described above.

### Statistical analysis and software.

Statistical analyses were performed for triplicate experiments using the two-tailed Student's *t* test (Prism 6 version 6.04). Figures were generated using Inkscape and Prism 8 version 8.0.2.

### Data availability.

The RNA-Seq data obtained in this study have been deposited in the Gene Expression Omnibus (GEO; https://www.ncbi.nlm.nih.gov/geo) with accession number GSE117911. In addition, all sequence reads were deposited in the NCBI Sequence Read Archive Database (SRA; https://www.ncbi.nlm.nih.gov/sra) and are associated with accession number PRJNA483555.
