# Supplementary figures and images for: Norovirus Replication in Human Intestinal Epithelial Cells Is Restricted by the Interferon-Induced JAK/STAT Signaling Pathway and RNA Polymerase II-Mediated Transcriptional Responses
Source: mBio. 2020 Mar 17;11(2):e00215-20. doi: 10.1128/mBio.00215-20 (PMC7078467; doi:10.1128/mBio.00215-20)

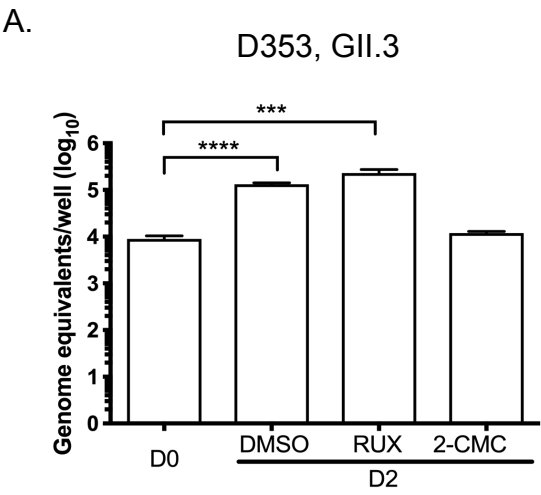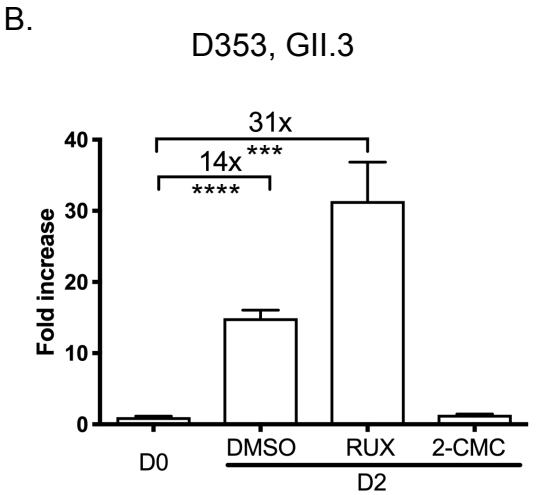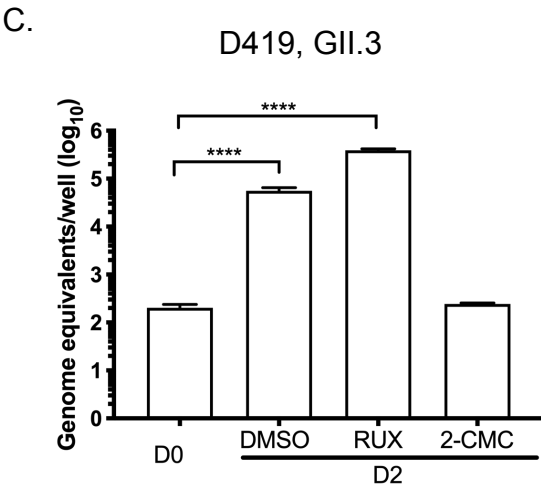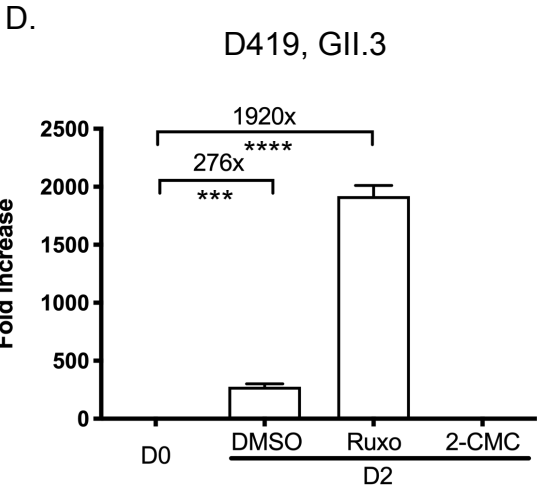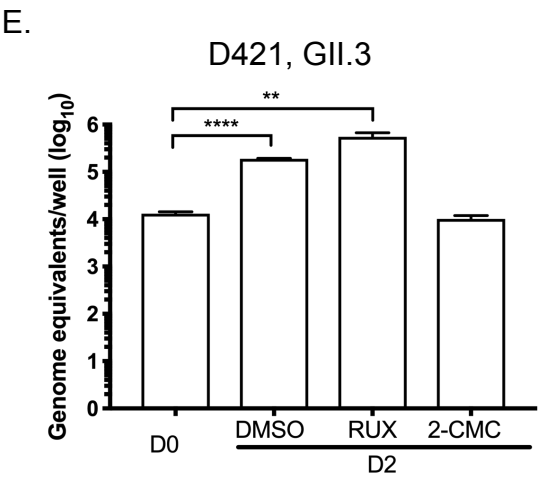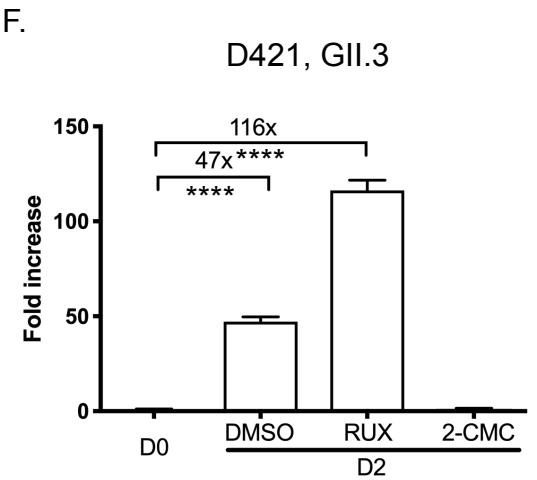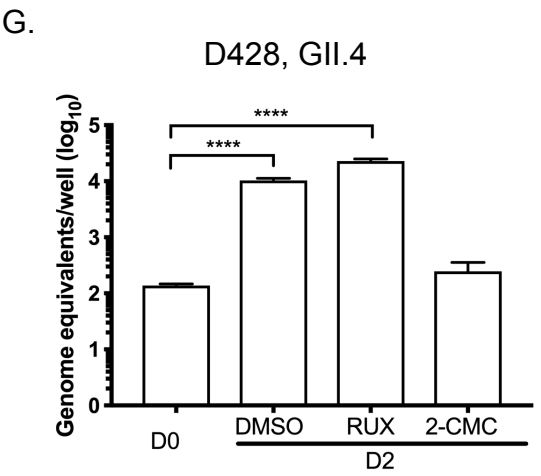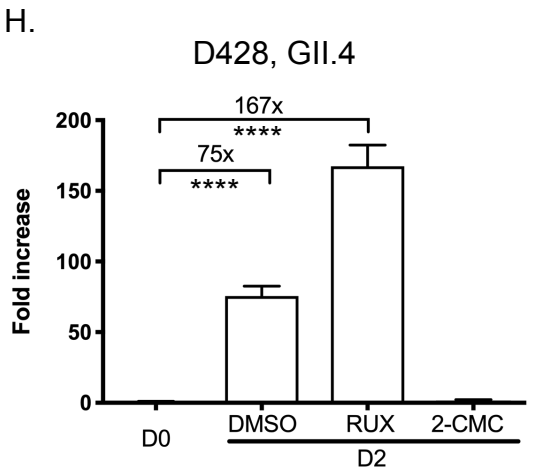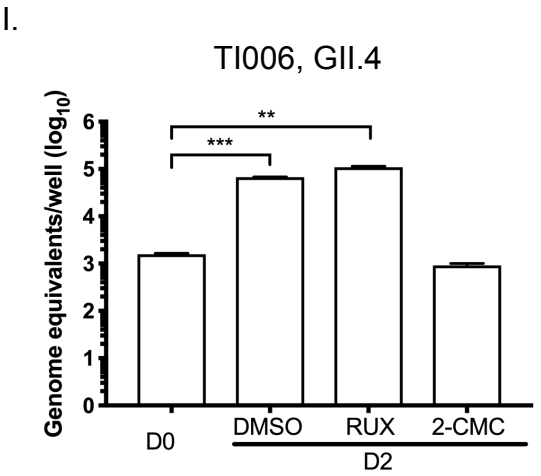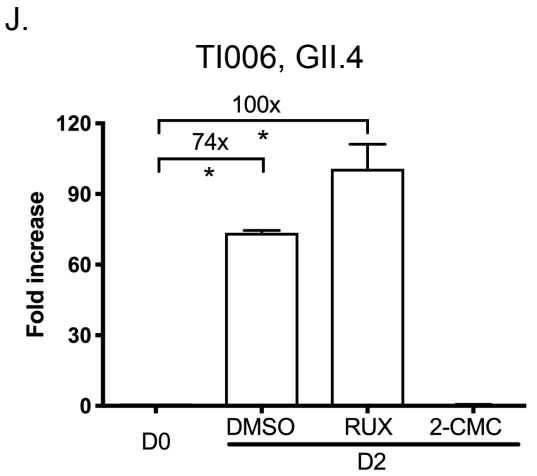

Supplement: FIG S1 [file mBio.00215-20-sf001.pdf]

A.

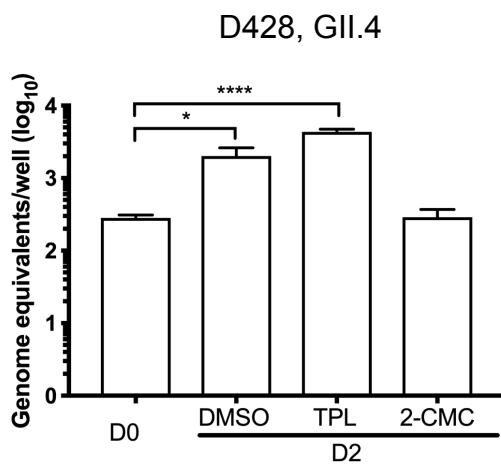

B.

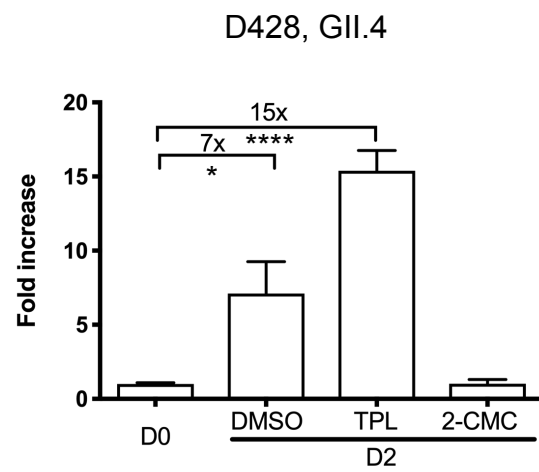

C.

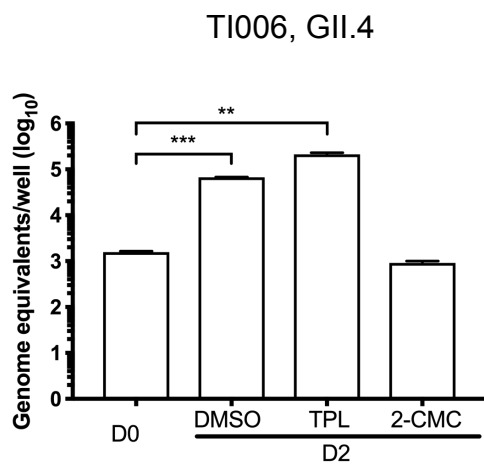

D.

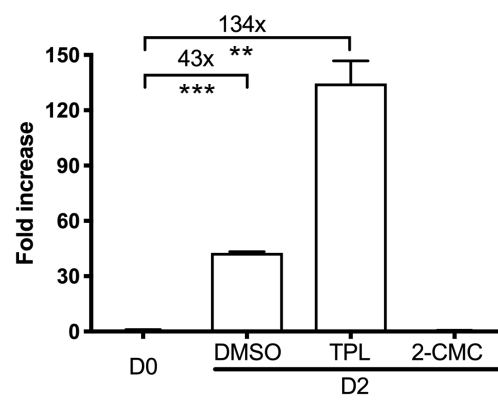

Supplement: FIG S2 [file mBio.00215-20-sf002.pdf]
